# Editorial: Learning in Times of COVID-19: Students', Families', and Educators' Perspectives

**DOI:** 10.3389/fpsyg.2022.915250

**Published:** 2022-05-18

**Authors:** Karin Gehrer, Sina Fackler, Karin Sørlie Street, Timo Gnambs, Ariel Mariah Lindorff, Kathrin Lockl

**Affiliations:** ^1^Leibniz Institute for Educational Trajectories (LIfBi), Bamberg, Germany; ^2^Deutsches Jugendinstitut, Munich, Germany; ^3^Department of Pedagogy, Religion, and Social Studies, Western Norway University of Applied Sciences, Sogndal, Norway; ^4^University of Oxford, Oxford, United Kingdom

**Keywords:** COVID-19, distance learning, home learning, student-teacher relationships, digital teaching and learning, learning

Whilst writing this editorial, we are looking back at almost 2 years of crisis due to the COVID-19-pandemic. From a first unprecedented lockdown in March 2020, after the first cases of this new virus disease were detected, to a series of more lockdowns, and hygiene regulations, it seems worthwhile to summarize findings that shed light on the situation of the education system. The present special issue on “*Learning in times of COVID-19: Students', Families', and Educators' Perspectives”* contains a collection of international empirical papers that analyze the situation of schoolteachers, pupils, university teachers, students, children, and parents. It offers insights into the situations of countries that had comparatively mild measures in place (e.g., Switzerland; cf. Garrote et al.; Helm and Huber) to countries that imposed weeks-long national lockdowns that completely isolated the country (such as Australia; cf. Martin et al.). Worldwide, parents had to juggle working from home while homeschooling or watching their children at the same time. Teachers and pupils had to move lessons online and get used to remote teaching formats. The same happened to university teachers and students around the world. Now, there is a generation of young people who have hardly seen their educational institution from the inside for the past 2 years and who, not to mention, suffered from severe contact restrictions that, in some cases, led to extreme social isolation. All of this was embedded in a situation of uncertainty regarding how the crisis would develop. The current special issue includes 40 research articles from all over the world that examined consequences of the pandemic in the educational context from multiple perspectives. Below, we present the articles according to four themes, pertaining to the situation of families, pupils, teachers and schools, and university students.

## On the Situation of Families

Families were under particular strain during the pandemic. They had to cope with home-schooling alongside home-office work (Canales-Romero and Hachfeld), and many parents reported feeling overburdened. Both social status and education level were related to how families were coping with the challenges of the pandemic (Sanrey et al.; Vogelbacher and Attig). Moreover, parents who had children attending primary as opposed to secondary school seemed to have been particularly affected (Garrote et al.).

Perhaps surprisingly, the findings of Canales-Romero and Hachfeld highlighted several positive effects of lockdowns for parents and families, with home-office-work positively affecting household dynamics and overall positive parental wellbeing. However, there were also increases in certain negative dynamics such as disputes. Taking on the role of “assistant teacher” in particular was a stressor, related negatively to household dynamics. The authors suggested implications for how schools communicate with and involve parents.

Sanrey et al. included preschool- to elementary-school-aged children and their parents in France to examine the risk of a digital divide during the COVID-19 lockdown. A higher social position was associated with a higher probability of owning more than one computer. At the same time, social position did not predict the time spent on computers to do schoolwork. The results revealed that nearly all parents were highly involved in setting up homeschooling for their children. However, parents with lower social position spent more time homeschooling their children and felt less able to support homeschooling, while also experiencing more fear about their children's academic failure.

Vogelbacher and Attig investigated predictors of parents' emotional stress and perceived abilities to support their children's learning during the first lockdown. Structural equation models demonstrated that these abilities were predicted by parents' level of education as well as previous perceived stress and socioeconomic status. Interestingly, higher-educated parents reported higher perceived stress during the COVID-19 lockdown.

Garrote et al. examined the relationship between parents' perceived threat of COVID-19 and their stress due to distance learning and their children's perceptions. As one of many interesting results they highlighted that parents of primary school students reported feeling more stressed than parents of secondary school students. Moreover, they found that female pupils experienced distance learning less positively than their male peers and felt more threatened by COVID-19.

Oppermann et al. investigated the role of parental support and home-learning environment on the provision of learning opportunities for 1–6 year-olds during day-care center lockdowns in Germany. Parental stress was negatively related to changes in the provision of home-learning environment (HLA). Parental self-efficacy and an intact social support system were protective factors against parental stress, alleviating the negative influence of stress on parents' ability to provide educational activities for their children at home. These results have important implications for supporting families with young children during challenging times.

## On the Situation of Pupils

During the pandemic primary and secondary school students were challenged by distance learning, but also by returning to alternate teaching in small groups (Thorsteinsen et al.). An important issue in this context is whether existing educational inequalities were increasing as a consequence of the pandemic (Berger et al.; Weber et al.; Zinn and Bayer). Several papers explored what factors may play a role in strengthening students' resilience and mental health (Dändliker et al.; Helm and Huber; Martin et al.) or students' satisfaction after lockdown (Li et al.). Also, the impact of students' cognitive and affective-motivational factors as resources in this time was investigated (Lockl et al.).

Dändliker et al. focused on the mental health of pupils in secondary education in the early phase of the pandemic and the role of perceived social support by teachers, friends, and parents. They identified three resilience-profiles that differed in terms of students' educational concerns and perceived family support. These criteria were also strong risk or protective factors during school closures.

Zinn and Bayer investigated potential changes in educational inequality as a result of the initial school closure by focusing on the time spent on school-related activities in German secondary schools prior to and during the pandemic. In support of their hypotheses, the authors found an initial equalization effect (i.e., students spent similar amounts of time on school-related activities regardless of their parents' education level) during the spring 2020 lockdown, followed by an increase in educational inequality after the lockdown. That is, in the period after the lockdown, students with lower educated parents spent less time on school-related activities as compared with students whose parents had higher educational attainment.

Thorsteinsen et al. described the challenges experienced by elementary school children in Norway after the schools reopened, when classes were divided into smaller groups. They reported that children who did not like their new group showed reduced emotional school engagement and subjective wellbeing.

The changes in adolescent satisfaction before and after lockdown were investigated by Li et al. in a professional adolescent sport training school in China. As a main result, they found that the satisfaction of adolescents improved significantly after the lockdown.

Predictors for students' learning outcomes in Austria and Switzerland were the focus of Helm and Huber. Students' ability to self-organize emerged as the most significant predictor across all three informant groups (pupils, parents, and teachers), while the lack of parental support during school closures turned out to be relevant only from the parents' perspective.

In a mixed-methods study, Sim et al. investigated children's and adolescents' coping with home learning and related contextual factors. Most children and adolescents perceived their coping with home learning as successful, and school joy before COVID-19, parental support, and available equipment during home learning predicted children's coping. Moreover, family climate, a quiet place to study, and also equipment were important for adolescents learning at home. Interviews showed that students applied individual strategies for coping with home learning, where family and peers had a vital role, especially when contact with teachers was limited.

Adopting the job demands-resources theory, Martin et al. investigated the role of adaptability (i.e., the capacity to adjust behaviors, thoughts, and feelings in response to unexpected circumstances) in helping Australian high school students navigate their online learning. The authors found that beyond the effects of online learning demands, online and parental learning support, and background attributes, adaptability was significantly associated with higher levels of online learning self-efficacy and with gains in later achievement. Online learning self-efficacy was also significantly associated with gains in achievement, and significantly mediated the relationship between adaptability and achievement. Consequently, the authors stressed the importance of adaptability as a personal resource in this process.

Investigating the longitudinal effects of distance schooling on existing educational inequalities of Austrian lower secondary school students, Berger et al. found a widening of the gap. Coping with out-of-school learning was especially challenging for students with low academic achievement and learning motivation prior to the pandemic. Furthermore, the findings demonstrated that support from parents and teachers fostered students' capabilities to cope with the self-regulatory demands connected with distance learning. The authors recommended strengthening self-regulation as an essential educational skill for academic achievement and life-long learning.

Using a within-person approach, Weber et al. investigated whether social and ethnic disparities in the reading achievement of Austrian primary school pupils widened during COVID-related school closures during spring 2020 and whether increased disparities were mediated by parental involvement in distance learning. Controlling for pre-lockdown reading differences, they found that low socioeconomic status and non-German language use at home negatively predicted post-lockdown reading achievement, indicating that post-lockdown disparities were larger than expected due to disparities at pre-lockdown. In contrast, they found no such effects during the pre-lockdown period. Second, a series of mediation models did not provide support for the hypothesis that parental involvement accounted for family background effects on reading achievement during the lockdown period.

Lockl et al. focused on cognitive and affective-motivational factors as possible predictors of coping with the demands of home learning in secondary school. Data from two measurement points from the German National Educational Panel Study (NEPS) revealed students' prior reading competencies and their willingness to exert effort as significant predictors, whereas other predictors (e.g., learning enjoyment, intrinsic motivation) had no effect on coping. Parents reported having more difficulties motivating children with lower reading competencies, or boys.

## On the Situation of Teachers and Schools

Teachers were suddenly challenged to change their teaching styles and methods and to adopt diverse digital tools. Some of the articles included in this section examined resilience-building factors (Schneider et al.; Spicksley et al.) or causes of teachers' stress (Colville et al.; Lindner et al.; Pöysä et al.). Moreover, the challenges experienced by teachers of students with special educational needs (SEN) in the distance-learning mode were explored (Maurer et al.), as well as how teacher training students plan to use digital learning materials in their future practice (Paetsch and Drechsel). Finally, some articles focused on the collaboration with parents such as how teachers made contact with parents (Hemmerich et al.) or how parents experienced teachers during this time (Haller and Novita).

Schneider et al. focused on a large sample of primary and secondary school teachers in Germany who reported on different aspects of distance teaching. The results highlighted the importance of regular contacts between the teachers and students during the remote learning period.

In England, Spicksley et al. did qualitative research on how teachers perceived their relationships with other teachers during the crisis and how psychological states (both negative and positive) were reported. They showed that teachers with a strong collective identity could better cope with the challenges than teachers lacking social support by their colleagues.

Lindner et al. considered the wellbeing of teachers in Austria during the pandemic, using an online survey over three waves. The teachers reported on their emotional experiences and job satisfaction before and after the first lockdown, and then in the second lockdown. Teacher job satisfaction was high overall but tended to decline during lockdowns. Cross-lagged path models showed interesting relationships between job satisfaction and positive and negative emotional activation over time. The authors highlighted the importance of addressing teachers' job satisfaction even after the pandemic has eased.

Hemmerich et al. investigated how and why professionals working in early childhood education and care (ECEC) centers in Germany did or did not make contact with parents during the lockdown. The authors found differences in the responses given according to different ECEC types, as well as according to the professionals' understanding of their own role. The authors discussed the importance of shared perceptions of responsibility among ECEC professionals, adequate digital tool training and support, and outreach strategies to connect with disadvantaged parents.

The importance of parents' perceptions of schools as a central indicator for assessing school quality was underlined in a study by Haller and Novita. During school lockdown, parents' school satisfaction may reflect schools' abilities to adjust and react to fast social changes with almost no time for preparation. Using longitudinal NEPS data they identified predictors of parents' perceptions of school support. The results suggested that parents were likely to be satisfied during school lockdowns when they had positive attitudes toward teachers prior to school lockdowns.

Colville et al. interviewed primary teachers in Scotland against the theoretical background of new engaged pedagogy. Teachers reported on changes in pedagogy, agile and flexible working, changing identities, and parental engagement.

The focus of Maurer et al. was on students with special educational needs (SEN). They investigated how students with SEN coped with the sudden distance learning, and whether teachers of students with SEN faced greater hurdles in handling this switch. The results revealed no significant differences between teachers of special schools and teachers of inclusive schools regarding the use of digital learning. All teachers reported being dissatisfied with more digital learning hours. A large part of distance learning was conducted off-line with worksheets, and books. Teachers' self-efficacy for distance learning was rather low for all teachers of students with SEN.

Paetsch and Drechsel examined teacher training and, in particular, how the first online semester in a German university contributed to pre-service teachers' intentions to use digital learning materials in the future. The quality of online instruction and self-reported improvements in digital skills were important factors in predicting students' intentions to use digital learning materials in the future. Different results were found for pre-service teachers training to work in elementary school vs. secondary school.

Pöysä et al. investigated latent profiles of Finnish primary school teachers' well-being and found four groups based on their occupational stress and work engagement. During the first few months of the COVID-19 pandemic many teachers experienced occupational stress as well as some increase in stress due to the pandemic. The findings provided new insights concerning how teachers' work engagement was, for some, not severely affected during the first few months of the pandemic, and on how different teaching styles were associated with different aspects of occupational well-being.

## On the Situation of University Students

Several contributions were dedicated to the experiences of university students and how they viewed the switch to distance learning (Goppert and Pfost; Guse et al.; Kovacs et al.; Mohr et al.). Other articles focused on the needs of these students to cope with this challenging time (Hopp et al.; Naujoks et al.; Teuber et al.). Teacher-student interactions, in particular warm relationships, were investigated as important factors in distance-learning (Capon-Sieber et al.; Sun et al.). Other articles developed a framework for online teaching (Wang et al.) or investigated synchronous vs. asynchronous settings of online teaching (Fabriz et al.). Variables explaining digital literacy (Hoss et al.), procrastination (Lim and Javadpour), or attitudes (e.g., usefulness) toward distance learning (Drueke et al.) and the emotional stress caused by conflicting information on the pandemic (Mayweg-Paus et al.) were also examined.

Kovacs et al. investigated the use of digital learning tools of Austrian university students before and during the first lockdown. The results showed that their use of classic digital media such as e-mail or chats did not change whereas the use of certain tools such as videos and web conferencing systems increased considerably. As students saw advantages as well as disadvantages of online learning vs. face-to-face-learning, the authors recommended a balanced combination of both approaches in future university teaching.

The effects of synchronous vs. asynchronous online teaching and learning settings in university were explored by Fabriz et al. They reported that students in predominantly synchronous online settings voiced greater satisfaction of their basic psychological needs for competence support and connectedness, as well as greater overall satisfaction with the online semester compared to students in predominantly asynchronous online settings.

Naujoks et al. addressed the important issue of self-regulated learning in a sample of university students in Germany. More specifically, they investigated students' digital readiness to cope with online learning as well as their intended and actual use of external resource management strategies. While students seemed to be prepared to study online, they were not able to manage their resources during the course as often as intended.

Teuber et al. examined students' psychological needs (i.e., autonomy or competence satisfaction) during the COVID-19 lockdown, their academic engagement or intention to drop out, and the relationship with institutional strategies (i.e., communication of the institution staff about the procedure of examinations and courses). The results emphasized the importance of timely information to students about the universities' strategies for examinations and courses as important institutional tasks during a crisis.

Mohr et al. addressed the requirements in medical studies in respect to their extensive practical components. This included benefits associated with digital learning such as flexibility for students with childcare or jobs and perceived disadvantages such as the lack of interactions with peers, professionals, and patients in practice. The study also explored term-specific effects as well as gender- and age-specific differences in students' satisfaction with the digital study program.

With a focus on the same target group, Guse et al. examined mental burden and study worries among undergraduate medical students. The study showed that a large proportion of medical students experienced significant levels of distress and mental burden during the COVID-19 pandemic. It also highlighted the need for ongoing psychological and educational support for this group of university students during the pandemic.

Goppert and Pfost explored the stress levels of German psychology students in summer 2020 compared to students in preceding academic terms. While a high or medium level of stress is common for university students, the change to e-learning seemed not to be stressful. Contrary to the assumptions, the results indicated that the students experienced fewer worries and more joy in their studies, although they had more workload on average.

The association between students' close social networks, digital information-sharing behavior, and their experiences of social and emotional loneliness during COVID-19 was investigated by Hopp et al. While digital information-sharing behavior, number of close online contacts, and interconnectedness and heterogeneity of contacts were associated with students' experiences of social loneliness, only the heterogeneity of close contacts was associated with students' experiences of emotional loneliness. The authors recommended universities offer training and support in easy-to-use communication software.

Sun et al. investigated the influence of teacher-student interactions on learning during online education in China, based on self-report questionnaires. The level of interaction between teachers and university students was positively related to learning, and the mechanism of this effect could be characterized *via* a chain-mediating effect such that teacher-student interactions affected psychological atmosphere, which affected learning engagement, which in turn affected academic performance. Given the foothold maintained by online teaching and learning even after lockdowns have been lifted, understanding such mechanisms is of critical importance globally, and in the long term.

Also set in China, Wang et al. drew on existing theory to develop a framework for measuring teaching presence in online teaching. Results showed good support for a five-factor model of teaching presence, including design and organization, facilitating discourse, direct instruction, assessment, and technological support.

Capon-Sieber et al. similarly considered the move from face-to-face to online courses in universities necessitated by the pandemic, and tested whether lecturers' support for relatedness drove student satisfaction with relatedness and, in turn, student motivation and vitality, with a moderating effect of affiliative motive (where a high affiliative motive reflects a wish for positive/warm relationships). The proposed mediation effect was evident but the moderating effect was not. Forms of communication (e.g., video chat) and class format (lecture/seminar) were both relevant to students' experiences of online learning.

Drueke et al. investigated the effect of the pandemic on online university education in Germany drawing on an extended version of the technology acceptance model. The authors showed that perceived usefulness and, to a lesser degree, perceived ease of use were the main predictors of attitudes toward distance learning. Moreover, the latter was associated with data security worries and the organization of online teaching, while the former was associated with general media affinity and pandemic-related worries.

In a sample of German master- and bachelor students, Hoss et al. found that learning opportunities and higher digital literacy depended on study progress and student characteristics. General self-efficacy, a private working space, anxiety, affect, age, and the perceived preparedness of lecturers for remote learning were identified as relevant variables explaining students' perceived probability of remote study success. The authors suggested that university students' digital literacy should be promoted early on.

In an online experiment, Mayweg-Paus et al. investigated the emotional stress caused by conflicting information on the pandemic. During discussions of textual information, participants in collaborative groups more often discussed the pandemic in general and less often engaged emotionally, as compared to individual responses. All participants reported higher perceived information overload, lower self-efficacy, and higher active coping strategies after the reflection task compared to before reading the information, with no significant differences between the collaborative groups and individuals. The authors stressed the importance of peer interaction and reflective skills when dealing with potentially stressful information.

Lim and Javadpour investigated effects on procrastination of university students across two semesters. They found that uncertainty and procrastination did not differ between prior to COVID-19 and the following semester. Uncertainty predicted procrastination, and students' life history strategy mediated the relation between uncertainty and procrastination. Uncertainty during the pandemic prompted students to psychologically shift their life history strategy such that it focused on present gains, which predicted procrastination.

## Methodological Approaches

A variety of approaches were evident in terms of methodology. Most of the research for this special issue was cross-sectional and emerged from researchers' rapid responses to the extreme changes. Some articles used an experimental (Mayweg-Paus et al.), qualitative, or mixed methods approach, or conducted in-depth interviews (e.g., Mohr et al.; Simm et al.). A few surveys relied on existing large longitudinal studies, for example longitudinal data from the German Socio-Economic Panel (SOEP) (Zinn and Bayer) or NEPS (Haller and Novita; Lockl et al.; Vogelbacher and Attig). However, most studies were set up in direct response to the lockdowns of education institutions and used online questionnaires, gathering information from around hundred participants to several thousands.

## Summary

As we can read in this special issue people have employed a myriad of coping strategies, but various psychological burdens have also been reported in relation to the COVID-19 pandemic. The special issue demonstrates how differently this international crisis has been dealt with, how it has affected different parts of society differently, but it has also brought to our attention a range of coping mechanisms. We thank all 145 authors and the around 100 reviewers for their great commitment to this important and timely topic. We hope that beyond the COVID-19 crisis, this special issue will be useful to practitioners as well as researchers, as it offers important insights based on rigorous academic research.

## Author Contributions

KG, SF, TG, and KL contributed to conception and design of the special issue and the editorial. SF wrote the first draft of the introduction of the editorial. KG, SF, KS, TG, AL, and KL wrote sections of the manuscript. All authors contributed to manuscript revision, read, and approved the submitted version.

## Conflict of Interest

The authors declare that the research was conducted in the absence of any commercial or financial relationships that could be construed as a potential conflict of interest.

## Publisher's Note

All claims expressed in this article are solely those of the authors and do not necessarily represent those of their affiliated organizations, or those of the publisher, the editors and the reviewers. Any product that may be evaluated in this article, or claim that may be made by its manufacturer, is not guaranteed or endorsed by the publisher.

